# 

**Published:** 1844-11

**Authors:** 


### CONTENTS

### OF NO. XI.

### ORIGINAL COMMUNICATIONS.

### ESSAYS AND CASES.

Art. I.—The Replier replied to, and the Reviewer reviewed; a letter to Lunsford P. Yandell, M.D., containing Strictures and Thoughts on the errors and false doctrines of Chemico-Physiology. By Carles Caldwell, M.D. - - - - - - - 373

Art. II.—An Essay on the Preparations of Iodine. By J. O'Ferrall, Jr., M.D., of Ohio. - - - - - 402

Art. III.—A Case of Extensive Emphysema, produced by a wound of the Trachea. By J. G. Girdner, of Galena, Illinois. - - - - - - - - - 417

### SELECTIONS FROM AMERICAN AND FOREIGN JOURNALS.

Progress of Surgery in China. - - - - - - - - 420

On the Cure of Hydrocele by Iodine Injections. - - 421

Fascination. - - - - - - - - - - - - - 422

Quarterly table of Mortality in Great Britain. - - - - 423

Influence of a Varying State of the Price of Provisions on General Disease and Mortality. - - - - - 423

The Local Pathology of Neuralgia. - - - - - - - 424

Flourine. - - - - - - - - - - - - - - 424

Extirpation of the Spleen. - - - - - - - 424
Calomel as a Remedy in Diseases of the Eye. - - - 425
Therapeutic Agency of Mesmerism. - - - - - - 425
Fresh Vaccine Lymph. - - - - - - - - 425
Spontaneous cure of Phthisis. - - - - - - - 426
Amenorrhœa cured by Electro-Galvanism. - - - 427
Comparative value of the different preparations of Mercury and Iodine, and the best modes of administering them. 428
Pain of the Loins. - - - - - - - - - 434
Effects of Camphor. - - - - - - - - 436
Antidotes of Corrosive Sublimate, Copper, Lead and Arsenic. 437
Brodie on Mercury in Syphilis. - - - - - - 438
Surgical Operations in Cancerous Diseases. - - - 440
Use of Colomba-Root against Vomiting. - - - - 441
Quackery. - - - - - - - - - - 441
Quinine against obstinate Hiccup. - - - - - 442
The Human Brain. - - - - - - - - 442
Psychological Medicine. - - - - - - - 443
Protective Influence of Vaccination. - - - - - 443
Combe on the Influence of Sleep in warding off Insanity. - 443
Useful Hints to Hospitals. - - - - - - - 444

ORIGINAL INTELLIGENCE.

Traveling Letter, No. VII. - - - - - - 445
Black-Tongue. - - - - - - - - - 451
Traveling Letter, No. VIII. - - - - - - 457
Professor Caldwell and Animal Chemistry. - - - 462
Dr. M'Dowell once more. - - - - - - - 463

